# Supplementary material for: Clinical utility of targeted SARS-CoV-2 serology testing to aid the diagnosis and management of suspected missed, late or post-COVID-19 infection syndromes: Results from a pilot service implemented during the first pandemic wave
Source: PLoS One. 2021 Apr 7;16(4):e0249791. doi: 10.1371/journal.pone.0249791 (PMC8026061; doi:10.1371/journal.pone.0249791)
Supplement: S1 Fig — Negative = 0: No visible borderline = 0.5: A visible band in ideal lighting conditions, positive = 1: A visible band in all lighting conditions, strong positive = 2: A visible band at the intensity of the control line or 3: A visible band of greater intensity than the control line. NB: Bands of 0.5 intensity are unable to be scanned/ photographed and therefore appear blank on the scanned image below. (DOCX) [file pone.0249791.s001.docx]

**S1 Fig.** Scanned images of LFIA cassettes for participants 084 (left) and 086 (right) labelled with band intensities. Negative = 0: no visible borderline = 0.5: a visible band in ideal lighting conditions, positive = 1: a visible band in all lighting conditions, strong positive = 2: a visible band at the intensity of the control line or 3: a visible band of greater intensity than the control line. NB: bands of 0.5 intensity are unable to be scanned/ photographed and therefore appear blank on the scanned image below.

**
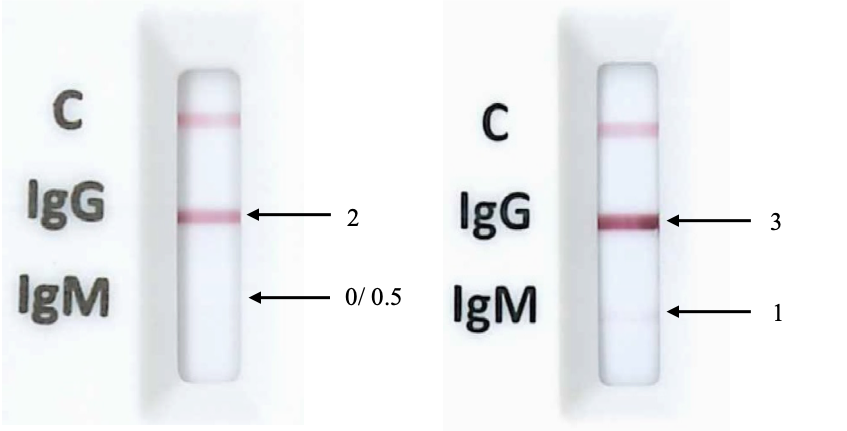
**
