# Supplementary material for: Clinical utility of targeted SARS-CoV-2 serology testing to aid the diagnosis and management of suspected missed, late or post-COVID-19 infection syndromes: Results from a pilot service implemented during the first pandemic wave
Source: PLoS One. 2021 Apr 7;16(4):e0249791. doi: 10.1371/journal.pone.0249791 (PMC8026061; doi:10.1371/journal.pone.0249791)
Supplement: S2 Fig — (DOCX) [file pone.0249791.s002.docx]

**S2 Fig:** flowchart of service delivery, same-day service, Monday to Friday.

Clinical team identify potential patient

Contact clinical virology service

Referral vetted

Sample identified for testing, logged and communicated to KCL laboratory team

Sample tested, image scanned, and result uploaded to Electronic Patient Record (EPR) with clinical comment

Recommendation for repeat testing at specified interval if clinically appropriate

Inappropriate indication

Clinical advice given

Appropriate indication

Antibodies NOT detected
