# Supplementary material for: Clinical utility of targeted SARS-CoV-2 serology testing to aid the diagnosis and management of suspected missed, late or post-COVID-19 infection syndromes: Results from a pilot service implemented during the first pandemic wave
Source: PLoS One. 2021 Apr 7;16(4):e0249791. doi: 10.1371/journal.pone.0249791 (PMC8026061; doi:10.1371/journal.pone.0249791)
Supplement: S3 Fig — (DOCX) [file pone.0249791.s003.docx]

**S3 Fig:** Cohort demographics including age, sex, category, direct care team, RNA result (if performed), SureScreen LFIA results (band intensity recorded from 0.5-3) and ELISA data (results expressed as fold change above background, ≥4 fold above background in either IgM or IgG is reported as positive).

| **Participant no.** | **Age** | **Sex** | **Category** | **Direct care team** | **Subcategory** | **SARS-CoV-2 RNA** | **SureScreen** | | | **ELISA (S)** | | | **ELISA (N)** | | |
| --- | --- | --- | --- | --- | --- | --- | --- | --- | --- | --- | --- | --- | --- | --- | --- |
|  |  |  |  |  |  |  | **IgM** | **IgG** | **Result** | **IgM** | **IgG** | **Result** | **IgM** | **IgG** | **Result** |
| 001 | 37 | F | Presentation associated with SARS-CoV-2 | Surgery | Ophthalmology | ND | 0.5 | 1 | Pos | 4.3 | 17.6 | Pos | 5.5 | 10.2 | Pos |
| 002 | 26 | M | Presentation associated with SARS-CoV-2 | Medicine | Nephrology | Neg | 0 | 0 | Neg | ND | | N/A | ND | | N/A |
| 003 | 58 | F | Presentation associated with SARS-CoV-2 | Medicine | Acute | Neg | 0 | 0 | Neg | 1.5 | 2.0 | Neg | 10.2 | 1.4 | Pos |
| 004 | 28 | F | Suspected ‘missed’ diagnosis of COVID-19 | Medicine | Respiratory | ND | 0 | 0 | Neg | 2.0 | 1.7 | Neg | 12.1 | 1.3 | Pos |
| 005 | 1 | F | Presentation associated with SARS-CoV-2 | Paediatrics | PIMS-TS | Pos | 0 | 3 | Pos | 5.3 | 24.5 | Pos | 5.5 | 10.3 | Pos |
| 006 | 76 | F | Suspected ‘missed’ diagnosis of COVID-19 | Medicine | Acute | Neg | 0 | 0 | Neg | 3.4 | 1.9 | Neg | 12.4 | 2.0 | Pos |
| 007 | 20 | M | Presentation associated with SARS-CoV-2 | Medicine | Intensive care | ND | 0.5 | 1 | Pos | 6.0 | 21.6 | Pos | 9.9 | 7.1 | Pos |
| 008 | 4 | M | Presentation associated with SARS-CoV-2 | Paediatrics | PIMS-TS | Neg | 1 | 3 | Pos | 8.2 | 24.2 | Pos | 7.4 | 15.5 | Pos |
| 009 | 40 | F | Suspected ‘missed’ diagnosis of COVID-19 | Medicine | ID/HIV | Neg | 0 | 0 | Neg | 1.8 | 1.3 | Neg | 6.0 | 1.3 | Pos |
| 010 | 0 | M | Presentation associated with SARS-CoV-2 | Paediatrics | Other | Neg | 0 | 0 | Neg | 0.7 | 1.3 | Neg | 1.7 | 1.2 | Neg |
| 013 | 9 | M | Presentation associated with SARS-CoV-2 | Paediatrics | PIMS-TS | Neg | 0 | 0.5 | Neg | 1.3 | 2.8 | Neg | 4.0 | 2.4 | Pos |
| 014 | 8 | F | Presentation associated with SARS-CoV-2 | Paediatrics | PIMS-TS | Neg | 0 | 0 | Neg | 2.3 | 1.1 | Neg | 4.1 | 0.7 | Pos |
| 015 | 6 | F | Presentation associated with SARS-CoV-2 | Paediatrics | PIMS-TS | Neg | 0 | 0 | Neg | 3.2 | 2.6 | Neg | 13.5 | 2.6 | Pos |
| 016 | 62 | M | Suspected ‘missed’ diagnosis of COVID-19 | Medicine | ID/HIV | Neg | 1 | 1 | Pos | 8.5 | 12.5 | Pos | 12.7 | 12.0 | Pos |
| 017 | 19 | F | Presentation associated with SARS-CoV-2 | Medicine | Cardiology | Neg | 0 | 0 | Neg | 2.2 | 1.5 | Neg | 6.3 | 1.1 | Pos |
| 018 | 6 | M | Presentation associated with SARS-CoV-2 | Paediatrics | PIMS-TS | Neg | 0 | 0 | Neg | 1.5 | 1.5 | Neg | 4.0 | 1.0 | Neg |
| 019 | 10 | M | Presentation associated with SARS-CoV-2 | Paediatrics | PIMS-TS | Neg | 0 | 0.5 | Neg | 2.1 | 12.9 | Pos | 2.0 | 5.9 | Pos |
| 020 | 61 | F | Suspected ‘missed’ diagnosis of COVID-19 | Medicine | Nephrology | ND | 1 | 2 | Pos | 10.1 | 22.0 | Pos | 10.9 | 15.4 | Pos |
| 021 | 61 | F | Suspected ‘missed’ diagnosis of COVID-19 | Medicine | Nephrology | ND | 1 | 2 | Pos | 12.1 | 22.7 | Pos | 15.9 | 15.2 | Pos |
| 022 | 7 | M | Presentation associated with SARS-CoV-2 | Paediatrics | Other | Neg | 0 | 3 | Pos | 3.8 | 22.3 | Pos | 5.2 | 11.3 | Pos |
| 023 | 45 | F | Suspected ‘missed’ diagnosis of COVID-19 | Medicine | Nephrology | Neg | 0 | 0 | Neg | 5.2 | 1.8 | Pos | 7.4 | 0.8 | Pos |
| 024 | 65 | M | Suspected ‘missed’ diagnosis of COVID-19 | Medicine | Nephrology | ND | 1 | 2 | Pos | 14.8 | 23.2 | Pos | 16.6 | 15.0 | Pos |
| 025 | 40 | F | Suspected ‘missed’ diagnosis of COVID-19 | Medicine | Nephrology | ND | 1 | 2 | Pos | 14.4 | 20.8 | Pos | 16.3 | 14.4 | Pos |
| 026 | 50 | M | Suspected ‘missed’ diagnosis of COVID-19 | Medicine | Nephrology | ND | 1 | 3 | Pos | 12.0 | 20.5 | Pos | 15.4 | 11.8 | Pos |
| 027 | 45 | M | Suspected ‘missed’ diagnosis of COVID-19 | Medicine | Nephrology | Neg | 0.5 | 1 | Pos | 10.4 | 17.2 | Pos | 4.2 | 3.8 | Pos |
| 028 | 53 | F | Suspected ‘missed’ diagnosis of COVID-19 | Medicine | Nephrology | ND | 0 | 0 | Neg | 2.3 | 1.6 | Neg | 10.5 | 1.5 | Pos |
| 029 | 14 | M | Presentation associated with SARS-CoV-2 | Paediatrics | PIMS-TS | Neg | 0.5 | 0 | Neg | 3.0 | 1.7 | Neg | 12.2 | 1.4 | Pos |
| 030 | 25 | F | Suspected ‘missed’ diagnosis of COVID-19 | Medicine | Nephrology | ND | 1 | 2 | Pos | 9.5 | 23.6 | Pos | 14.3 | 15.2 | Pos |
| 031 | 14 | F | Presentation associated with SARS-CoV-2 | Paediatrics | PIMS-TS | ND | 1 | 3 | Pos | 6.9 | 22.7 | Pos | 7.7 | 14.0 | Pos |
| 032 | 55 | F | Suspected ‘missed’ diagnosis of COVID-19 | Medicine | Intensive care | Neg | 1 | 3 | Pos | 13.2 | 23.6 | Pos | 17.1 | 15.3 | Pos |
| 033 | 5 | M | Presentation associated with SARS-CoV-2 | Paediatrics | PIMS-TS | Neg | 0 | 3 | Pos | 2.8 | 18.1 | Pos | 4.0 | 12.1 | Pos |
| 034 | 12 | M | Presentation associated with SARS-CoV-2 | Paediatrics | PIMS-TS | Neg | 0 | 0 | Neg | 2.4 | 1.3 | Neg | 5.4 | 1.8 | Pos |
| 035 | 64 | F | Suspected ‘missed’ diagnosis of COVID-19 | Medicine | Respiratory | Neg | 2 | 2 | Pos | 13.7 | 21.4 | Pos | 15.2 | 14.6 | Pos |
| 036 | 11 | F | Presentation associated with SARS-CoV-2 | Paediatrics | PIMS-TS | Neg | 0 | 2 | Pos | ND | | N/A | ND | | N/A |
| 037 | 59 | M | Suspected ‘missed’ diagnosis of COVID-19 | Medicine | Nephrology | Neg | 1 | 1 | Pos | 14.7 | 22.7 | Pos | 12.6 | 7.0 | Pos |
| 038 | 46 | F | Suspected ‘missed’ diagnosis of COVID-19 | Medicine | Nephrology | ND | 0 | 0 | Neg | 2.1 | 1.2 | Neg | 3.8 | 0.8 | Neg |
| 039 | 33 | M | Presentation associated with SARS-CoV-2 | Medicine | Intensive care | Neg | 1 | 2 | Pos | 8.5 | 22.2 | Pos | 8.1 | 13.2 | Pos |
| 040 | 3 | M | Presentation associated with SARS-CoV-2 | Paediatrics | PIMS-TS | ND | 0 | 0 | Neg | 2.8 | 1.3 | Neg | 11.6 | 1.2 | Pos |
| 041 | 73 | M | Suspected ‘missed’ diagnosis of COVID-19 | Medicine | Nephrology | Neg | 1 | 1 | Pos | 14.2 | 16.7 | Pos | 13.4 | 14.9 | Pos |
| 042 | 55 | F | Suspected ‘missed’ diagnosis of COVID-19 | Medicine | Nephrology | ND | 0.5 | 3 | Pos | 11.9 | 22.1 | Pos | 14.9 | 6.3 | Pos |
| 043 | 36 | F | Suspected ‘missed’ diagnosis of COVID-19 | Medicine | Respiratory | ND | 0 | 0 | Neg | 1.9 | 2.1 | Neg | 6.3 | 1.1 | Pos |
| 044 | 44 | F | Suspected ‘missed’ diagnosis of COVID-19 | Medicine | Acute | Neg | 0 | 0 | Neg | 2.1 | 1.2 | Neg | 5.5 | 0.9 | Pos |
| 045 | 56 | F | Presentation associated with SARS-CoV-2 | Medicine | Respiratory | Neg | 0 | 0 | Neg | 5.4 | 1.7 | Pos | 4.1 | 8.0 | Pos |
| 046 | 36 | M | Presentation associated with SARS-CoV-2 | Medicine | Cardiology | Neg | 0 | 0 | Neg | 3.6 | 13.4 | Pos | 1.5 | 6.4 | Pos |
| 047 | 4 | F | Presentation associated with SARS-CoV-2 | Paediatrics | PIMS-TS | Neg | 0 | 0 | Neg | 3.3 | 1.5 | Neg | 5.8 | 1.3 | Pos |
| 048 | 48 | F | Suspected ‘missed’ diagnosis of COVID-19 | Medicine | Respiratory | ND | 0.5 | 0.5 | Pos | 11.4 | 15.9 | Pos | 3.4 | 11.1 | Pos |
| 049 | 71 | F | Suspected ‘missed’ diagnosis of COVID-19 | Medicine | Respiratory | ND | 0 | 3 | Pos | 18.5 | 23.5 | Pos | 10.2 | 14.9 | Pos |
| 050 | 30 | F | Suspected ‘missed’ diagnosis of COVID-19 | Medicine | Respiratory | Neg | 0 | 0 | Neg | 7.2 | 1.0 | Pos | 5.6 | 0.9 | Pos |
| 051 | 33 | F | Suspected ‘missed’ diagnosis of COVID-19 | Medicine | Respiratory | Neg | 0 | 0 | Neg | 4.1 | 1.1 | Pos | 8.5 | 1.5 | Pos |
| 052 | 69 | F | Suspected ‘missed’ diagnosis of COVID-19 | Medicine | Nephrology | ND | 0 | 0 | Neg | 2.6 | 1.1 | Neg | 1.7 | 0.9 | Neg |
| 053 | 49 | M | Presentation associated with SARS-CoV-2 | Medicine | Respiratory | Neg | 0 | 0 | Neg | 2.4 | 1.8 | Neg | 1.9 | 2.6 | Neg |
| 054 | 53 | M | Suspected ‘missed’ diagnosis of COVID-19 | Medicine | Respiratory | Neg | 0 | 0 | Neg | 1.4 | 1.0 | Neg | 8.4 | 0.6 | Pos |
| 055 | 16 | M | Presentation associated with SARS-CoV-2 | Paediatrics | PIMS-TS | Neg | 0 | 1 | Pos | 3.9 | 2.8 | Neg | 4.4 | 2.8 | Pos |
| 056 | 11 | F | Presentation associated with SARS-CoV-2 | Paediatrics | PIMS-TS | Neg | 0 | 2 | Pos | 10.1 | 20.9 | Pos | 6.1 | 27.5 | Pos |
| 057 | 12 | M | Presentation associated with SARS-CoV-2 | Paediatrics | PIMS-TS | Neg | 0 | 0 | Neg | 2.7 | 2.2 | Neg | 3.8 | 2.3 | Neg |
| 058 | 14 | M | Presentation associated with SARS-CoV-2 | Paediatrics | PIMS-TS | Neg | 0.5 | 3 | Pos | 12.8 | 22.3 | Pos | 4.1 | 1.9 | Pos |
| 059 | 64 | M | Suspected ‘missed’ diagnosis of COVID-19 | Medicine | Respiratory | Neg | 1 | 2 | Pos | 23.7 | 23.1 | Pos | 12.3 | 15.5 | Pos |
| 060 | 83 | M | Suspected ‘missed’ diagnosis of COVID-19 | Medicine | Respiratory | Neg | 0.5 | 3 | Pos | 19.2 | 24.4 | Pos | 10.9 | 15.2 | Pos |
| 061 | 12 | M | Presentation associated with SARS-CoV-2 | Paediatrics | PIMS-TS | Neg | 0 | 2 | Pos | 8.3 | 20.4 | Pos | 2.4 | 12.4 | Pos |
| 062 | 28 | F | Suspected ‘missed’ diagnosis of COVID-19 | Medicine | Respiratory | ND | 1 | 0 | Pos | 10.8 | 6.5 | Pos | 4.8 | 6.2 | Pos |
| 063 | 33 | M | Suspected ‘missed’ diagnosis of COVID-19 | Medicine | Acute | Neg | 0 | 0 | Neg | 2.7 | 1.1 | Neg | 2.6 | 1.4 | Neg |
| 064 | 11 | F | Presentation associated with SARS-CoV-2 | Paediatrics | PIMS-TS | Neg | 0 | 0 | Neg | 5.0 | 1.7 | Pos | 6.1 | 1.2 | Pos |
| 065 | 12 | F | Presentation associated with SARS-CoV-2 | Paediatrics | PIMS-TS | Neg | 0 | 0 | Neg | 3.9 | 1.2 | Neg | 2.9 | 0.8 | Neg |
| 066 | 51 | F | Suspected ‘missed’ diagnosis of COVID-19 | Medicine | Nephrology | Neg | 1 | 3 | Pos | 23.5 | 22.5 | Pos | 9.8 | 13.3 | Pos |
| 067 | 10 | F | Presentation associated with SARS-CoV-2 | Paediatrics | PIMS-TS | ND | 1 | 3 | Pos | 11.1 | 23.9 | Pos | 4.6 | 12.0 | Pos |
| 068 | 10 | F | Presentation associated with SARS-CoV-2 | Paediatrics | PIMS-TS | Neg | 0 | 0 | Neg | 4.7 | 1.8 | Pos | 4.6 | 1.5 | Pos |
| 069 | 35 | F | Presentation associated with SARS-CoV-2 | Medicine | ID/HIV | Neg | 0 | 0 | Neg | 4.8 | 1.1 | Pos | 6.8 | 1.0 | Pos |
| 070 | 4 | M | Presentation associated with SARS-CoV-2 | Paediatrics | PIMS-TS | Neg | 0.5 | 3 | Pos | 16.9 | 23.7 | Pos | 5.7 | 14.8 | Pos |
| 071 | 4 | F | Presentation associated with SARS-CoV-2 | Paediatrics | PIMS-TS | Neg | 0 | 0 | Neg | 3.2 | 0.9 | Neg | 2.8 | 1.5 | Neg |
| 072 | 7 | F | Presentation associated with SARS-CoV-2 | Paediatrics | PIMS-TS | Neg | 0 | 0 | Neg | 2.0 | 1.7 | Neg | 3.0 | 1.4 | Neg |
| 073 | 9 | F | Presentation associated with SARS-CoV-2 | Paediatrics | Other | Neg | 0 | 0 | Neg | 2.6 | 1.1 | Neg | 2.6 | 0.9 | Neg |
| 074 | 28 | F | Presentation associated with SARS-CoV-2 | Medicine | Respiratory | ND | 0 | 0 | Neg | 4.8 | 3.0 | Pos | 6.5 | 2.4 | Pos |
| 075 | 15 | F | Presentation associated with SARS-CoV-2 | Paediatrics | Other | Neg | 0 | 0 | Neg | 4.9 | 2.9 | Pos | 3.9 | 1.3 | Neg |
| 076 | 71 | F | Suspected ‘missed’ diagnosis of COVID-19 | General Practice |  | ND | 0.5 | 1 | Pos | 5.6 | 18.8 | Pos | 12.2 | 15.5 | Pos |
| 077 | 40 | M | Suspected ‘missed’ diagnosis of COVID-19 | General Practice |  | ND | 0.5 | 1 | Pos | 13.6 | 7.8 | Pos | 7.1 | 12.2 | Pos |
| 078 | 47 | M | Presentation associated with SARS-CoV-2 | Medicine | ID/HIV | Neg | 1 | 2 | Pos | 21.0 | 23.3 | Pos | 6.9 | 11.2 | Pos |
| 079 | 37 | M | Presentation associated with SARS-CoV-2 | Medicine | Acute | Neg | 0 | 0 | Neg | 1.3 | 1.4 | Neg | 1.5 | 0.9 | Neg |
| 080 | 73 | F | Presentation associated with SARS-CoV-2 | Medicine | Haematology | Neg | 0 | 0 | Neg | 1.7 | 0.8 | Neg | 1.6 | 0.9 | Neg |
| 081 | 72 | F | Presentation associated with SARS-CoV-2 | Medicine | Haematology | Neg | 0 | 0 | Neg | 4.2 | 1.5 | Pos | 3.9 | 1.2 | Neg |
| 082 | 56 | M | Suspected ‘missed’ diagnosis of COVID-19 | Medicine | Intensive care | Neg | 1 | 3 | Pos | 14.5 | 23.9 | Pos | 2.5 | 14.1 | Pos |
| 083 | 71 | F | Presentation associated with SARS-CoV-2 | Medicine | Haematology | Neg | 0 | 0 | Neg | 1.8 | 3.3 | Neg | 1.8 | 1.9 | Neg |
| 084 | 32 | F | Presentation associated with SARS-CoV-2 | Medicine | Acute | Neg | 0.5 | 2 | Pos | 8.6 | 10.8 | Pos | 8.6 | 11.4 | Pos |
| 085 | 64 | M | Presentation associated with SARS-CoV-2 | Surgery | Urology | Neg | 0 | 0 | Neg | 1.3 | 0.8 | Neg | 1.3 | 1.5 | Neg |
| 086 | 72 | M | Presentation associated with SARS-CoV-2 | Medicine | Respiratory | Neg | 1 | 3 | Pos | 12.0 | 12.1 | Pos | 12.0 | 14.8 | Pos |
| 087 | 55 | M | Suspected ‘missed’ diagnosis of COVID-19 | Medicine | Respiratory | Neg | 0 | 0 | Neg | 1.2 | 0.9 | Neg | 1.2 | 1.4 | Neg |
| 088 | 10 | M | Presentation associated with SARS-CoV-2 | Paediatrics | Other | Neg | 0 | 0 | Neg | 1.8 | 1.0 | Neg | 1.8 | 1.7 | Neg |
| 089 | 5 | M | Presentation associated with SARS-CoV-2 | Paediatrics | PIMS-TS | ND | 0 | 0 | Neg | 2.0 | 0.8 | Neg | 2.0 | 1.3 | Neg |
| 090 | 1 | F | Presentation associated with SARS-CoV-2 | Paediatrics | PIMS-TS | Neg | 0 | 0 | Neg | 2.0 | 1.2 | Neg | 2.0 | 1.8 | Neg |
| 091 | 4 | M | Presentation associated with SARS-CoV-2 | Paediatrics | PIMS-TS | Neg | 0 | 0 | Neg | 2.3 | 1.6 | Neg | 2.3 | 2.6 | Neg |
| 093 | 82 | F | Infection control/ immunosuppression management | Medicine | Oncology | Pos | 0 | 2 | Pos | 7.1 | 12.1 | Pos | 7.1 | 14.1 | Pos |
| 094 | 67 | F | Presentation associated with SARS-CoV-2 | Medicine | Respiratory | Neg | 0 | 0 | Neg | 1.4 | 1.0 | Neg | 1.4 | 1.9 | Neg |
| 095 | 20 | M | Suspected ‘missed’ diagnosis of COVID-19 | Medicine | Intensive care | Neg | 0 | 0 | Neg | 1.3 | 0.6 | Neg | 1.3 | 1.1 | Neg |
| 096 | 74 | M | Suspected ‘missed’ diagnosis of COVID-19 | Medicine | Oncology | ND | 0 | 0 | Neg | ND | | N/A | ND | | N/A |
| 097 | 82 | M | Infection control/ immunosuppression management | Medicine | Oncology | Pos | 1 | 2 | Pos | 14.7 | 11.9 | Pos | 14.7 | 13.9 | Pos |
| 098 | 61 | M | Suspected ‘missed’ diagnosis of COVID-19 | Medicine | Acute | Neg | 1 | 1 | Pos | 4.7 | 1.0 | Pos | 4.7 | 1.2 | Pos |
| 099 | 22 | M | Infection control/ immunosuppression management | Medicine | Nephrology | Pos | 1 | 2 | Pos | 12.0 | 10.5 | Pos | 12.0 | 13.3 | Pos |
| 100 | 62 | M | Infection control/ immunosuppression management | Medicine | Nephrology | Pos | 2 | 3 | Pos | 15.3 | 12.3 | Pos | 15.3 | 14.4 | Pos |
| 101 | 53 | M | Infection control/ immunosuppression management | Medicine | Nephrology | Pos | 2 | 2 | Pos | 14.4 | 10.8 | Pos | 14.4 | 14.0 | Pos |
| 102 | 64 | F | Infection control/ immunosuppression management | Medicine | Nephrology | Pos | 2 | 1 | Pos | 15.0 | 10.7 | Pos | 15.0 | 14.4 | Pos |
| 103 | 35 | F | Suspected ‘missed’ diagnosis of COVID-19 | Medicine | Obstetrics | ND | 0 | 1 | Pos | 2.4 | 8.2 | Pos | 2.4 | 5.0 | Pos |
| 104 | 30 | F | Presentation associated with SARS-CoV-2 | Medicine | Dermatology | ND | 0 | 0 | Neg | 2.0 | 0.7 | Neg | 2.0 | 1.0 | Neg |
| 105 | 11 | M | Presentation associated with SARS-CoV-2 | Paediatrics | PIMS-TS | Neg | 0 | 0 | Neg | 1.6 | 0.7 | Neg | 1.6 | 1.1 | Neg |
| 106 | 22 | M | Presentation associated with SARS-CoV-2 | Medicine | Respiratory | Neg | 0 | 0 | Neg | 1.2 | 1.0 | Neg | 1.2 | 1.7 | Neg |
| 107 | 30 | F | Presentation associated with SARS-CoV-2 | Medicine | Dermatology | ND | 0 | 0 | Neg | 2.0 | 1.0 | Neg | 2.0 | 1.3 | Neg |
| 108 | 32 | F | Presentation associated with SARS-CoV-2 | Medicine | Dermatology | ND | 0 | 0 | Neg | 1.5 | 1.0 | Neg | 1.5 | 1.5 | Neg |
| 109 | 31 | M | Presentation associated with SARS-CoV-2 | Medicine | Dermatology | ND | 0 | 0 | Neg | 3.4 | 1.0 | Neg | 3.4 | 2.0 | Neg |
| 110 | 64 | M | Presentation associated with SARS-CoV-2 | Medicine | Acute | Neg | 0 | 0 | Neg | 1.3 | 1.2 | Neg | 1.3 | 1.2 | Neg |
| 111 | 54 | M | Presentation associated with SARS-CoV-2 | Medicine | Acute | Neg | 0 | 0 | Neg | 1.5 | 0.8 | Neg | 1.5 | 1.7 | Neg |

ND = not done, Neg = negative, Pos = positive, N/A = not applicable
