# Supplementary material for: Clinical utility of targeted SARS-CoV-2 serology testing to aid the diagnosis and management of suspected missed, late or post-COVID-19 infection syndromes: Results from a pilot service implemented during the first pandemic wave
Source: PLoS One. 2021 Apr 7;16(4):e0249791. doi: 10.1371/journal.pone.0249791 (PMC8026061; doi:10.1371/journal.pone.0249791)
Supplement: S4 Fig — Defined dilutions of the NIBSC research reference reagent for anti-SARS-CoV-2 antibody (20/130) were tested in triplicate on the SureScreen LFIA. Results are displayed as a heat map, with white indicating a negative result and gradations of orange representing the magnitude of response detected. (DOCX) [file pone.0249791.s004.docx]

**S4 Fig.** IgG limit of detection for the SureScreen LFIA. Defined dilutions of the NIBSC research reference reagent for anti-SARS-CoV-2 antibody (20/130) were tested in triplicate on the SureScreen LFIA. Results are displayed as a heat map, with white indicating a negative result and gradations of orange representing the magnitude of response detected.

**
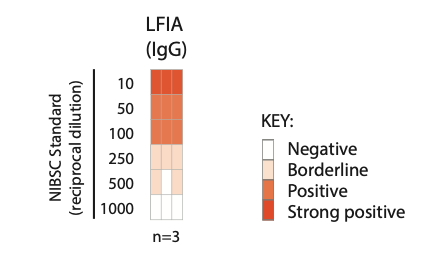
**
