# Supplementary material for: Clinical utility of targeted SARS-CoV-2 serology testing to aid the diagnosis and management of suspected missed, late or post-COVID-19 infection syndromes: Results from a pilot service implemented during the first pandemic wave
Source: PLoS One. 2021 Apr 7;16(4):e0249791. doi: 10.1371/journal.pone.0249791 (PMC8026061; doi:10.1371/journal.pone.0249791)
Supplement: Attachment — Submitted filename: Response to Reviewers 1.docx [file pone.0249791.s005.docx]

Dear Editor,

We have made the necessary amendments to the manuscript, as per the journal requirements. Please find below our rebuttal to the Reviewers.

General comment in response to Reviewers

We accept there has been progress with COVID-19 diagnostics since this study was done. The clinical need has also changed as the pandemic has evolved. However, this prospective pilot LFIA serology service was introduced shortly after the first COVID-19 wave and with comprehensive in-house validation. There was significant diagnostic uncertainty for patients presenting with a variety of clinical syndromes and needs, particularly given absence of community PCR testing. There was nationally co-ordinated implementation of serology testing using core technology platforms (Roche, Abbott) that could not meet this need, in part because they recognise nucleocapsid and not spike proteins. There were also changing MHRA requirements making it challenging to meet laboratory validation requirements to safely introduce a service.

The strengths and limitations of this study have been more clearly presented in this context and we have updated the abstract introduction and discussion to reflect these changes. We propose that this will address the major concerns of reviewers relating to the study design and setting.

We therefore believe that with these changes this study deserves publication in PLOS ONE and these fall into three main reasons.

1) It describes a range of clinical presentations of post-COVID-syndromes and clinical scenarios where serology can aide diagnosis. This is important data to be published after peer review. There have been theoretical descriptions (e.g. <https://doi.org/10.1136/bmj.m3325>) of the potential clinical utility and diagnostic challenges in different prevalence settings, but not real-life data that this study provides.
2) LFIAs can still have value in settings that do not have accessible laboratory support providing more advanced flow cytometry and ELISA tests. This paper provides independent validation data conforming to MHRA requirements, information on how to report results including loading of results onto electronic systems and clinical pilot data to support their assessment for introduction in such settings.
3) We published the basic science work (Seow J et al Nature Micro PMID: 33106674) and technology evaluation research (Pickering S et al PLOS Path PMID: 32970782). This paper presents the final validation, governance approval and pilot evaluation stages to complete full description of a 3 month end-to-end pathway from discovery to service. There is therefore wider benefit in presenting completion of a translational research pathway in a pandemic setting. Indeed, the description of what went well and what didn’t can help inform improvements in diagnostic development pathways during a pandemic for the future.

Response to main concerns of Reviewer #1

1) In a previously published study (Plos pathogen (2020) Pickering et al.) the authors already extensively validated/compared LFIA devices including the commercially available SureScreen LFIA. Therefore, the comprehensive LFIA validation shown in figure 1 re-produces their own initially published data but is not new. This also raises the question of whether another patient cohort has been used for the analysis shown in this study. Additionally, raw data points and group clustering strategy are missing.

**Response:**

*The data is new as presented and indicated in the methods (expanded from initially being in the introduction).*

“The SureScreen LFIA was selected for further validation based on results from previous head-to-head analyses (Pickering et al 2020), provision of additional proprietary information on the antigen target by the manufacturer and confidence in procurement.”

*Pickering et al compared multiple technologies with a limited sample set. During our service implementation planning stage the regulatory requirements for laboratory validation of serological technologies increased such that we needed to provide results on a larger cohort of samples to meet MHRA and therefore local governance requirements.*

2) The sensitivity of the LFIA test in table 1b has also already been described in the earlier study (Plos pathogen, Pickering et al. 2020, Figure 5a).

**Response:**

*Given we needed to perform a larger validation before introducing the service we presented updated sensitivity and specificity in the paper.*

3) The high sensitivity (accuracy) of the test might derive from the biased sample group which is 14 and 20 days POS (Peak of AB response) and would rather not reflect the “real-life” situation in clinics. Therefore, clinical interpretation of results might rather be difficult (no quantification of the AB response) if random samples should/will be tested and interpreted. This is further highlighted in the study of Pickering et al. (Plos pathogen (2020) where the sensitivity (of all tests, including SureScreen) is highly dependent on the “days after onset of symptoms”.

**Response:**

*Our study was done in real-life where a decision to test was made on a case-by-case basis, which is one of its strengths. It was delivered with clinical oversight where the limitations described by the reviewer would be considered before agreeing to perform the tests and before communicating the result. This is how we propose LFIA tests could still continue to have clinical utility in some settings. Not as a high throughput/random sample service without clinical details where there would be more concerns about time of sample collection from symptom onset.*

4) Quantification and interpretation of antibody dynamics is not possible with this assay. Furthermore, additional data on IgA levels in nasopharyngeal swaps would be helpful and informative and would strengthen the considered serology service in clinics.

**Response:** *we agree that nasopharyngeal IgA levels would strengthen serology services in clinics and that prospective studies need to be reported.*

5) In addition to the LFIA limitations described above, serology only identifies a proportion of recently infected patients within a short time frame. Thus, seronegative results (more than 60% of the cases, table 2) are difficult to interpret and resulting interventions as well as management would be extremely difficult and speculative with this test. CD4+/CD8+ T cell memory assays might close the gap (Peng et al.2020, Nature immunology).

**Response**:

*We agree that serology is likely to be positive within a short time-frame. It was that knowledge that prompted setting up this service in May 2020 using what we considered the best available option at that time. Indeed there were no formally recommended technologies for patient testing. Since then studies have been conducted on the utility of T cell responses that may close the gap although they presumably will be tests in specialist laboratories with technology such as flow cytometry. We have recognised the limitations of negative serology in the discussion.*

6) Split of patient samples into different pathologies (Table 1a) is fairly interesting but loses its statistic power (small sample size).

**Response**

*We did not intend to conduct this study to perform statistical analysis. The intention was to offer the test in response to the breadth of specialties requesting antibody testing. We therefore think the data as presented best fits the study design.*

7) “Neutralising antibody titers” are missing from the neutralising antibody assay described in methods and results.

**Response**

*We have added the ID50 in for the six participants.*

8) Within this small cohort, rational for testing and the resulting interpretation is highly speculative and demonstrate rather a case report.

**Response**:

*The scenario as presented was real life responding to a clinical need. The rational for testing came from a discussion in each case between the referring clinician and the clinical virologist. We don’t think it is highly speculative, we think it presents useful data at a specific point in the translational research pathway.*


Response to minor concerns of Reviewer #1

1) Study design (flow chart) would be helpful to follow

**Response**:

*We have included a flowchart as Supplementary figure 1.*

2) Age characteristics of individual patients would be helpful

**Response**

*We respectfully disagree that including this level of individual patient detail would not add to the overall message of the manuscript, and therefore have not included. We provided summary statistics (median [IQR]) and do have the individual ages if the Editor wants them.*

3) The SureScreen LFIA was selected based on which criteria?

**Response**

*The SureScreen was selected from the initial evaluation because it was one of 3 with acceptable performance criteria, the manufacturer openly provided their proprietary information that we could review and we had dependable UK procurement route, which at that time was a major consideration. We have updated the methods to indicate this.*

*None of the authors had any personal conflicts of interest with SureScreen, although they did provide the kits for free specifically for this pilot service and after we had selected them.*

4) How did you exactly correlate/categorize LFIA results with ELISA data into the different categories as “negative”,“borderline”, “positive” and “strong positive”?

**Response**

*Negative – no visible band on LFIA; less than 3-fold above background for ELISA*

*Borderline – a faint/weak band; 3-4.99-fold above background in ELISA*

*Positive – a clear, visible band; 5-9.99-fold above background in ELISA*

*Strongly positive – a strongly positive band, greater in intensity than the control band; 10-fold or more above background in ELISA.*

*Results were based on 2 independent reviewers. We have now added that to the methods.*

5) Neutralising antibody titers for 6 individuals with persistent SARS-CoV-2 are missing

**Response**

*We have now included these.*

6) Can you run blood samples on the LFIA and do those results correlate with serum samples (much faster processed) on the LFIA? Which dilution is required (serum/blood)?

**Response**

*We did not use whole blood in this study but accept that this should be done alongside assessment such as dilution. For both PCR testing (nose, nose and throat, nasopharyngeal) and serology testing (serum, whole blood, pin-prick) there is considerable debate about which samples to use and a lot of comparative evaluation work is required. Collecting such new samples prospectively now would be very difficult and is a large project in its own right. We therefore propose that data is not required for this publication.*

7) Rapid decline in AB titers of SARS-CoV-2 infected patients is controversially discussed and should be interpreted carefully.

**Response**

*We have removed this from the updated version of the manuscript.*

Response to main concerns of Reviewer #2

1) In the Methods and Results section, the authors mention the assessment of Limit of Detection, but there are no Results shown. Visualization of these experiments would help to interpret the heat-map results shown in figure 1. Are these LoD dilutions mentioned in the results for IgM or IgG?

**Response**

*The LoD dilutions results (for IgG only as the reference material was a convalescent sample from a patient who had recovered from COVID-19) are now included.*

2) How are the 168 samples in fig. 1 selected?

**Response**

*This is a random selection of samples from hospital inpatients admitted to Guy’s and St Thomas’ hospitals during the first wave of the pandemic with a variety of disease severities and for which symptom onset was known. We have now made that clear in the results.*

3) Table 1b: are the values for sensitivity and specificity for IgG, IgM or a combination of both?

**Response**

*Sensitivity and specificity are based on a positive result from either IgM or IgG as per the manufacturer’s instructions presented in the methods.*

4) Are borderline results calculated as positive or negative?

**Response**

*Borderline bands were reported as positive in the evaluation of specificity and sensitivity as per the manufacturer’s instructions. For the purposes of the pilot a repeat test at a specified interval was recommended for confirmation if a borderline band was seen for only IgM or IgG.*

5) It is difficult to understand the sensitivity and specificity values, as 5 of 19 sera at d14 are negative. Are these samples representative?

**Response**

*The sensitivity calculations are for all samples 14+ (plus) days post onset of symptoms. Therefore samples at 14 days post onset of symptoms represent only a small proportion of the entire sample set.*

6) I propose that the results of the pilot study are presented in the same manner as the results in Figure 1, i.e. strength of reaction for IgG and IgM, alongside with the time point after POS.

**Response**

*Only the binary nature of the result impacted on clinical management i.e. antibodies detected, or not detected. Therefore we respectfully disagree that this is not required, although we could of course include it if requested by the Editor.*

7. Paragraph 3 of results: the indications of % in brackets are misleading, as the 29 pediatric patients with PIMS-TS are 100%, the following numbers should be adapted accordingly.

**Response**

*We have removed the percentages to reduce confusion*.

8) In the same paragraph are 7 patients mentioned, but only 6 described – what about the last patient?

**Response:**

*We only describe 6 patients. We have made that clearer, so that it will not be interpreted as there being a 7^th^ patient*

9) Paragraph 5 of results: the implication drawn by the authors is not very robust. There is no evidence for this implication, as results are lacking. Is there correlation between the threshold cycle of RNA detection and the strength of Antibody signal in the LFIA?

**Response**

*We do not think that correlating threshold RNA cycle and antibody will help. The antibody results are semi-quantitative and a CT value of >30 was taken as a threshold at which the individual was unlikely to be infectious.*

Furthermore, the results of the neutralization experiments should be included in the comparison of RT-PCR and LFIA results for these follow-up patients.

**Response**

*We have included the neutralisation titres in the updated manuscript.*

10) The authors claim, that this pilot should address the question, whether the serological rapid antibody test may help in the clinical routine in decisions. As mentioned in the methods, the clinicians using the service described, have been contacted for informal feedback and their view of utility. These feedbacks are not systematically evaluated.

**Response**

*We have clarified the method we used to gather informal feedback. We did not actively contact clinicians to get their feedback and so could not perform systematic feedback and so this has been clarified in the methods*

11) Paragraph 5 of discussion: the first sentence does not help a lot.

**Response**

*We have improved this sentence as part of improving the overall presentation*

12) Paragraph 5 of discussion: how are the inclusion criteria set, to obtain high pre-test probability? Could you explain how the PPV and NPV with the different pre-test probabilities are calculated?

**Response**

*The inclusion criteria for any individual patient were not set with fixed criteria because the purpose of the study was to assess the range of requests received. At that time there were no recommendations on who to test. Thus the decision to test a patient was based on the clinical judgement of the virologist after discussing individual patients with the referring clinician. The PPV and NPV were calculated based on a prevalence of 45% that was the percentage positives we saw in the sample cohort. In effect this is a post-hoc measure of the decision of whether to test or not. It demonstrates it was being provided to an appropriate population of patients*

This concludes our rebuttal.

Yours sincerely,

Blair Merrick
