# Supplementary material for: Clinical utility of targeted SARS-CoV-2 serology testing to aid the diagnosis and management of suspected missed, late or post-COVID-19 infection syndromes: Results from a pilot service implemented during the first pandemic wave
Source: PLoS One. 2021 Apr 7;16(4):e0249791. doi: 10.1371/journal.pone.0249791 (PMC8026061; doi:10.1371/journal.pone.0249791)
Supplement: Attachment — Submitted filename: Reponse to Reviewers.docx [file pone.0249791.s006.docx]

RESPONE TO REVIEWERS

Reviewer #1: I carefully read the manuscript and the corresponding responses to the raised concerns to both reviewers and noted (at least for my section) that these points are not sufficiently addressed as well as important raw data sets are still missing.
I therefore do not support direct publication at Plos one based on the following concerns.

*Response: We appreciate reviewer 1 putting significant time into this review. We have now addressed these concerns as best we can to further improve the work. This includes adding further data and toning down our conclusions where we have overstated what is possible from a lateral flow assay. We also referenced other now-published data including the comparative assays we have performed. We believe we have sufficiently addressed these points now to justify publication.*

Concern 1: The LFIA validation in figure 1, as discussed in the first revision, has not been satisfactory addressed. In general, the “Pickering study” validated +/- 50 samples less. Therefore, the reasoning of a limited dataset in the “Pickering study” or an “extended” data set in this study is not valid in this case.

*RESPONSE: We considered it necessary to present this “extended” dataset at the start (perhaps alternatively as a supplementary figure) because that is what was required by MHRA at the time. MHRA initially published requirements for 100 +ve and –ve tests, and then increased to 200 +ve and –ve samples. We were not in a position to judge the validity of this decision or the wider criteria, we just had to follow the request to gain internal approval to provide the pilot service and in preparation for any external scrutiny that might appear. We have made reference to that in the methods, results and discussion.*

Furthermore, requested raw data (ELISA data, LFIA pictures, neutralization assay setup with appropriate controls) are missing in the .

*RESPONSE:*

1) *We have provided the raw ELISA data in a supplementary figure and performed additional IgM experiments which were not reviewed previously.*
2) *We have created a supplementary figure showing the band intensity for 0/0.5, 1, 2, 3.*
3) *For neutralisation assay set we have referenced our PIMS-TS paper (*[*https://www.nature.com/articles/s41591-020-1054-6*](https://www.nature.com/articles/s41591-020-1054-6)*)*

Concern 2: Responses to concern 2/3 has only been partially addressed. Even though the manuscript includes “a bigger dataset” the current test validation strategy (samples 14+ POS) do still not reflect the “real-life” test-sensitivity from my point of view.

*RESPONSE: we are a translational research laboratory based in the clinical diagnostic virology laboratory. All these samples were collected prospectively in real time from every identifiable patient as they were admitted and longitudinally during their stay. We used all available samples for validation and point out that tests are performed and results interpreted based on considering the relevant clinical data. We also conducted each test only after initial clinical discussion. We do not recommend the test is done as an automated test request without clinical oversight. We therefore think this is “real-life” and represents the end to end process from test validation through to service delivery, rather than a laboratory validation stage considered alone. We accept that there will be different perspectives of what “real-life” means, but consider ours is an acceptable one.*

The authors claim that they consider this limitation before agreeing to perform the test. Can you include potential POS of these patients in the manuscript, to get a better feeling how to interpret these negative LFIA tests (+/- 60%) in figure 2?

*RESPONSE: Yes we have highlighted that these tests were performed between 21 and 90 days POS. We consider that if samples are going to be antibody positive they will almost certainly have been so by 21 days. From our other work looking at longevity of antibody response, we would still expect to detect a serological response at 90 days POS.*

Concern 3: Response to concern 3 has only been partially addressed since comments on the AB quantification and AB dynamics has not been discussed in the manuscript. AB dynamics might represent an important and useful service implementation when included in the service (not only for borderline results) and should be included in the manuscript.

*RESPONSE: We have now referenced our paper on antibody dynamics (*[*https://www.nature.com/articles/s41564-020-00813-8*](https://www.nature.com/articles/s41564-020-00813-8)*) where the assay and data are described in detail rather than represent published data. We have also added a comment on dynamics to direct readers.*

Concern 4 [Quantification and interpretation of antibody dynamics is not possible with this assay]- has only been partially addressed in the discussion.

*RESPONSE: We accept that quantification and antibody dynamics is not possible with this assay. Again, we have now referenced our paper on antibody dynamics (*[*https://www.nature.com/articles/s41564-020-00813-8*](https://www.nature.com/articles/s41564-020-00813-8)*). We also make a statement in the discussion of why we provided the semi-quantitative test result but also that we only provided “Detected” and “Not-Detected” in the clinical result.*

Concern 5: has not been addressed since the Specificity (95% CI) describes a statistical test and the data are at least linked/interpreted like this (Title of the table: Specificity of SureScreen LFIA). The authors should tone down their statements or adjust the table.

*RESPONSE: We have adjusted the table [figure 2a and 2b] accordingly and have toned down statements in the text.*

Concern 6: has not been addressed. Even though ID50 values were now reported in the results neither an experimental layout with appropriate controls nor plaque pictures or raw data from these assays are included in the manuscript. Implementation of this experiment is necessary to interpret the results and will strengthen the manuscript.

*RESPONSE: We have presented more detail on the experimental layout and controls used (by referencing previous work -*[*https://www.nature.com/articles/s41591-020-1054-6*](https://www.nature.com/articles/s41591-020-1054-6)*). We did not keep the plaque pictures and have not been asked for such data in any of our other publications and cannot see why that or further raw data for this clinical paper is required.*

Concern 7: Minor concern 4 is partially addressed. It is hard to believe how those bands can be grouped by eye in the corresponding categories. Moreover, I have not seen any pictures of those bands yet in the manuscript. Can you please provide those LFIA images? Quantification of these pictures would be helpful to categorize those samples into the corresponding group.

*RESPONSE: This is a very valid point that we can see will help readers and those considering introduction of lateral flow assays into service. We have presented these data as pictures and brought a better description of the semi-quantification method up into the beginning of the methods. People performing these tests rapidly become experienced such that independent reading of the LFIAs reaches good concordance which can be seen with the pictures now presented.*

*Since this study was conducted it is also of note that as increasing numbers of clinical staff, patients and public are having SARS-CoV-2 antigen tests performed, and the concept of strong and weak positive bands are presented in the guidance documents. We also report band strength in our work on antigen LFDs that has been submitted for publication (*[https://www.medrxiv.org/content/10.1101/2021.02.27.21252427v1](https://scanmail.trustwave.com/?c=8248&d=65TA4MwxBZ1LoQK_8VlLuphGX5ygPUTCWRbpaymfDw&u=https%3a%2f%2fwww%2emedrxiv%2eorg%2fcontent%2f10%2e1101%2f2021%2e02%2e27%2e21252427v1)).  *There will need to be greater clarity on the utility (or not) of recording and communicating band strength assessed by eye. Presentation of the data at this stage with appropriate caveats will help inform this debate.*

Concern 8: Minor concern 4 [How did you exactly correlate/categorize LFIA results with ELISA data into the different categories as “negative”,“borderline”, “positive” and “strong positive”?] is not addressed. See above

*RESPONSE: See response to concern 7 plus we reviewed our response to minor concern 4 in discussion with the scientists and they endorse the ease and consistency obtained with the categories. We therefore stand by it and have gone on to include in the methods so that the criteria on which we created the results can be considered by readers as below*

*Negative (0) – no visible band*

*Borderline (0.5) – a visible band in ideal lighting conditions*

*Positive (1) – a visible band in all lighting conditions*

*Strong positive (2/3) – a visible band at the intensity (2) or greater intensity (3) of the control band*

Concern 9: Minor concern 6 has not been addressed. If there is one potential niche for LFIA tests then at locations with limited lab material. Capillary blood instead of serum preparation could dramatically simplify the test procedure. Therefore, including those Blood/Plasma validations on the LFIA would indeed strengthen the manuscript.

*RESPONSE: Capillary blood testing would indeed dramatically simplify the test procedure (although limited laboratory material would only need to be a centrifuge and cabinet which should be available in any small laboratory). During this study a Medical Device Agency alert was published raising concern about capillary kits saying they were not reliable (June 20^th^ 2020). (*[*https://www.cas.mhra.gov.uk/ViewandAcknowledgment/ViewAlert.aspx?AlertID=103052*](https://www.cas.mhra.gov.uk/ViewandAcknowledgment/ViewAlert.aspx?AlertID=103052)*)*

*This illustrates the rapidly changing environment in which such studies were conducted. We therefore did not at the time progress to this comparison (beyond an initial evaluation) and cannot easily go back to do what would be a large piece of work. We think this is now something for future study with more developed POCT systems.*

Reviewer #2: The following questions have not been addressed properly.
Q2. It is not visible in the Results section, that these 168 Samples have been randomly selected.

*RESPONSE: This has been added to the results section.*

Q8. the authors still mention 7 out of 33 PCR negative patients, but describe only 6 patients. This point has not been clarified.

*RESPONSE: We have added details for the omitted patient.*

Q11. this question has not been addressed in the revised manuscript. The changes are not visible.

*RESPONSE: We have removed this sentence*

Please note that all data has been reanalysed as part of this resubmission, and with this there have been some minor changes to the values reported in this updated (compared to the original) manuscript.

We have also added an additional source of funding that was omitted in the original manuscript.
